# Management of Anaesthesia for a High-Risk Aerosol-Generating Procedure in a Paediatric Patient with COVID-19

**DOI:** 10.1155/2021/5568725

**Published:** 2021-03-19

**Authors:** Gezy Giwangkancana, Ezra Oktaliansyah, R. Ayu Hardianti Saputri

**Affiliations:** ^1^Department of Anaesthesiology and Intensive Care, Faculty of Medicine, Universitas Padjadjaran or Dr Hasan Sadikin National Referral Hospital, Bandung, Indonesia; ^2^Department of Otorhinolaryngology Head and Neck Surgery, Faculty of Medicine, Universitas Padjadjaran or Dr Hasan Sadikin National Referral Hospital, Bandung, Indonesia

## Abstract

**Introduction:**

Paediatric patients represent a small portion of the COVID-19 disease population. Nevertheless, the possibility of a paediatric patient requiring surgery, especially high-risk aerosol-generating surgery on the airway, while having the SARS-CoV-2 infection may potentially result in problems during the perioperative period due to concerns regarding patient, family, and staff safety. When unplanned and unrehearsed, this scenario may cause delays and efficiency issues. Our aim is to report on an 8-year-old patient with a foreign object lodged in the oesophagus with COVID-19 that required emergency surgery. *Case Report*. An 8-year-old female patient came to the emergency room with a history of difficulty in swallowing for 12 hours before admission, having accidentally swallowed a metal coin while playing. She did not have any recent history of disease, but her parents had noticed that, for the previous 4 days, she had had a mild fever and dry cough. Her parents and other relatives in the house had no similar complaints, and they assured us they had not been in contact with any suspected or confirmed COVID-19 patients. Our goal was to create a safe paediatric anaesthesia environment with safe working conditions for the surgical team. In this case report, we will describe our approach to patient transport, parental presence, preventions of aerosol risk, personal protection, the anaesthesia induction technique, and postoperative management.

**Conclusion:**

Safe paediatric anaesthesia, especially in a high-risk aerosol-generating procedure, during the COVID-19 era requires consideration and preparation of both the patient and healthcare provider. Multidisciplinary team work with an emphasis on a systematic and planned approach is required to improve efficiency.

## 1. Introduction

COVID-19 emerged as a pandemic at the end of 2019 with more than 50 million cases worldwide and 1 million fatalities by November 2020 [1]. Commonly affecting adult patients, paediatric patients have represented a very small group within the disease population, with less than 10% of global COVID-19 cases occurring within the paediatric age group. In addition, the fatality rate is low in the paediatric population. Asymptomatic cases have proven common in children, although a number have developed severe respiratory symptoms. The fatality rate is higher among the paediatric population with the presence of coexisting diseases such as heart anomalies, immunodeficiency, and cancer [1–3].

Providing surgery during the pandemic requires careful consideration regarding patient and staff safety. Greater resources and an altered workflow had to be developed to cater for this particular need. Anaesthesia in a paediatric patient is widely recognised as a challenging issue for the anaesthesiologist, and the addition of COVID-19 may complicate matters more, especially when the case involves the airway manipulation that increases aerosol generation and the potential for infection of the surgical team. The aim of this study is to describe the anaesthesia and operation room management for a high-risk aerosol-generating procedure in a paediatric patient with COVID-19 for the extraction of a foreign body by esophagoscopy.

## 2. Case Report

An 8-year-old girl weighing 27 kg referred from the city of Cirebon in West Java was admitted to our emergency room with difficulty in swallowing. The patient claimed that she had accidently swallowed a coin twelve hours before admission and, although still able to drink, had since then felt nauseous with constant pain in her throat. She did not have any previous history of disease, but her family had noticed that, for the previous 4 days, she had had a mild fever and dry cough. Her parents and other relatives in the house had no similar complaints, and they assured us they had not been in contact with any suspected or confirmed COVID-19 patients. Cirebon at that time was in an area considered a low risk for COVID-19.

**Figure 1:**
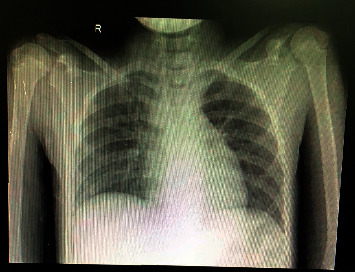
Radiological examination showing a foreign body in the cervical-7 to thoracic-1 area.

Upon physical examination, the child seemed well but showed signs of mild dehydration such as dry lips and tongue. She was fully alert, and her temperature was 36.7 C, her respiratory rate was 24 breaths per minute, her heart rate was 94 beats per minute, and lung auscultation was unremarkable. There were no inspiratory stridor, suprasternal, epigastric, or intercostal muscle retractions. The laboratory results showed slight leukopenia of 6.290 *µ*l with an unremarkable neutrophil count of 64%, and the absolute lymphocyte was 1700 *µ*l with a neutrophil-lymphocyte ratio of 2.37. A chest X-ray presented an image of a circular foreign object between the cervical-7(C7) to thoracic-1(Th1), possibly in the oesophagus, with no signs of pneumonia (Figure 1). As part of routine screening, a rapid antibody test for SARS-CoV-2 using a serum blood sample (kit: Vivadiag) was carried out, and the results were positive for IgM but negative for IgG. A real-time polymerase chain reaction (RT PCR) nasopharyngeal swab test was positive for SARS-CoV-2 with a cycle time (CT) helicase ORF1ab 33.74 gen RDRP 36.98 (kit: MbiocoV). The entire process from admission to the start of surgery was for 14 hours. The patient was consulted for rigid esophagoscopy and foreign body extraction. The COVID-19 surgical personnel were notified and assembled in the COVID-19 surgical theatre wing.

The preoperative preparation was performed simultaneously in the designated COVID-19 operation suite (COVID-19-OR) that occupies one wing of the central operating theatre. The anaesthesia team consisted of one attending anaesthesiologist, one resident, and one anaesthesia nurse. The anaesthesia team started routine anaesthesia preparation including preparation of the anaesthesia machine, monitors, suctions, and drugs with additional preparations not carried out in routine paediatric anaesthesia. The anaesthesia machine, anaesthesia monitors, video laryngoscopy monitor, bed surface, working table, work space, and all additional equipment were covered with a premade plastic wrap. The prepared drugs were labelled using a large coloured marker for easy identification, and disposable video laryngoscope blades were prepared for intubation. The team prepared povidone iodine 1% gargling solution and saline for the child to gargle with.

During this period, the surgical team prepared the instruments and the pharmacist prepared 12 sets of personal protection equipment, employing checklists on the 12 changing stations in the hallway. The nurse in charge added boots and face shields with coded names for easy staff identification. The nurse in charge also prepared communication sets such as a telephone, two handy talk sets, and in-room closed-circuit television (CCTV) systems.

After the surgical and anaesthesia setups were completed, the entire team changed into level-three personal protection equipment in a dressing hall outside the operating theatre. The donning was conducted using a checklist and buddy system in a designated dressing station for each of the personnel. This entire process lasted 35 minutes. As the patient was transferred to the operating theatre, the team conducted a time-out procedure. The difference from the routine surgical time-out was that the nurse in charge also reminded the team of the exit methods, disrobing and shower protocols, and staff symptom monitoring after surgery.

The patient was anxious, so the aunt was requested to accompany her to the COVID-19-OR. The aunt was given an N-95 mask and a face shield for protection. The patient was transported to the operating theatre using a specialized transport tube to prevent contamination of our hospital hallways, and a disinfectant crew was assigned to spray disinfectant solution as the transport tube passed by. The patient was received in the COVID-19-OR admission area by the anaesthesia team while other team members waited in the dressing area and suited up. The child, with help from her aunt, was asked to wash her mouth and gargle with the prepared betadine solution inside an aerosol tube. The child was given 0.5 mg of intravenous midazolam as premedication so she could be calmly separated from her aunt and was taken to the operation room.

The monitor was attached, and the patient's blood pressure was recorded at 100/70 mmHg, heart rate at 77 beats per minute, respiratory rate at 22 breaths per minute, and her saturation level was 97% on room air. The child was preoxygenated with 100% oxygen with a tightly sealed mask using the two-hand technique and was given 75 micrograms of fentanyl as premedication. Rapid sequence induction was carried out with 60 mg of propofol and 25 mg of rocuronium. The airway was secured with a 5.5 nonkinking endotracheal tube, and anaesthesia was maintained with sevoflurane 2–4 vol% in 50 : 50% air and oxygen mixture. The ventilator settings noted were the tidal volume at 180–200 cc, peak pressure of 14–16 mmH_2_O, and saturation at 95–97%.

**Figure 2:**
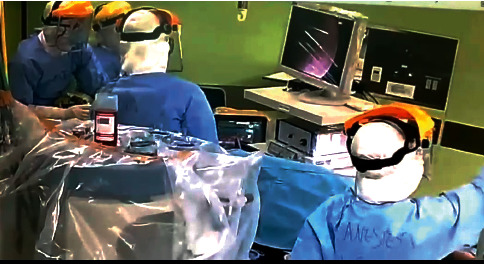
Foreign body extraction process.

**Figure 3:**
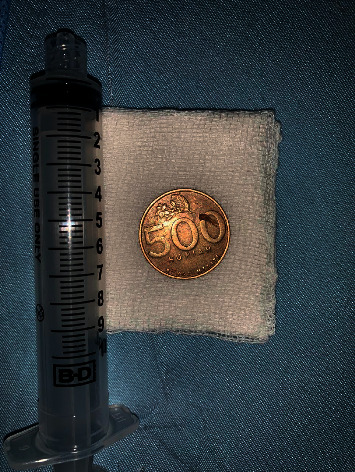
Foreign body extracted.

The surgical team entered the operating room after the patient had been intubated and an esophagoscopy set had been prepared. Upon visualization with endoscopy and esophagoscopy on the proximal part approximately 17 cm from the rim of the tooth to the oesophagus, the metal coin was located and was extracted using forceps (Figures 2 and 3). The surgical procedure lasted 15 minutes.

At the end of surgery, the child was given a bolus of fentanyl and extubated. The team began disrobing protocols one by one starting from the anaesthesiologist, surgeon, and surgical team. After the standard discharge criterion had been met, the anaesthesia resident and the nurse took the patient out of the COVID-19-OR and passed her on to the in-patient COVID-19 nurse waiting in the admission area for further care in the COVID-19 in-patient facility.

The patient was treated for 24 days in the COVID-19 unit with no further complaints. Interestingly, repeat RT-PCR showed a decreasing cycle threshold (Ct) (Table 1), and on the 19^th^ day, her antibody was reactive at 1.4. Swabs of her caretakers were all negative. Since she was being cared for by her aunt in the hospital, on the 5^th^ day of her admission, the aunt tested positive for COVID-19 though she remained asymptomatic and continued to provide care for the patient.

## 3. Discussion

To our knowledge, there have been limited reported cases of anaesthesia for high-risk aerosol-generating procedures on paediatric COVID-19 patients below the age of 10 years. Studies have found that only 10 percent of COVID-19 patients are children, and the odds against a COVID-19 patient requiring airway surgery make this case stand out [1–3]. We realize that this is a single case and data extrapolated from it may not be applicable to the general population.

This patient was asymptomatic on arrival which is in concordance with studies that found 94.1% of paediatric patients with Covid19 were asymptomatic on admission [3]. This child also did not have any contact history and lived in a relatively low-risk area of the country. Laboratory results did not show known COVID-19 characteristics such as low lymphocyte counts or abnormalities in a chest X-ray [2–4]. Although the clinical findings in this case report were similar to other studies, other modalities such as a chest computed tomography may provide a better correlation with a positive PCR. Studies show a high percentage of asymptomatic COVID-19 patients have abnormalities in the lungs, such as ground glass opacities, patchy shadows, and consolidations [4, 5].

It is possible that the laboratory values and chest X-ray were normal in relation to the higher CT value of the patient. Studies have shown that the cycle threshold (Ct) values of RT-PCR have some correlation with a viral load, infectivity, and disease severity where lower values < 24 may have worse disease severity [6, 7]. However, this theory is still debatable since our patient also remained asymptomatic even after a lower Ct value was recorded during isolation (Table 1). It is possible that the severity of the disease is not only mainly related to the viral load but also related to the patient's overall health status and comorbidity. This finding suggests that, in countries and areas where COVID-19 prevalence is high and there is a very real possibility of asymptomatic cases arriving in the operation theatre and with no other test as sensitive and specific for SARS-CoV-2, an obligatory swab should be carried out preoperatively when possible.

The only relevant test was a positive rapid antibody test which was useful in this case. Although the WHO does not support the use of rapid antibody testing in a clinical setting, it may be useful for early screening. Further study is needed on the relevance of rapid antibody tests in the preoperative period [8].

Since the surgery was related to airway manipulation, we used 1% povidone iodine mouth solution for preoperative gargling to reduce the risk of transmission to health-care workers in the operating theatre, especially since this was an airway surgery where aerosol-generating procedures were more likely. Studies have found that antiseptic mouth rinses, such as those containing methylpyridinium chloride or povidone iodine, may work to decrease the severity of COVID-19 by reducing the oral viral load in infected subjects and decreasing the risk of transmission by limiting the viral load in droplets generated in daily activity or in aerosol procedures such as intubation and airway related surgery [9, 10]. This approach has been the standard procedure for all of our surgical patients prior to anaesthesia induction, but further study is required regarding its effectivity.

Compared to our experience in performing surgery on adult patients with COVID-19, anaesthesia for paediatric patients with COVID-19 has presented us with some potential issues to resolve. Firstly, the child could not be taken to the operating theatre without a guardian, and sedation in the emergency room prior to transfer was deemed impossible due to the possibility of respiratory depression and a need for ventilation during transfer. Secondly, the child was already very anxious due to the unusual isolation conditions and was crying a lot which potentially generated more aerosols. Thirdly, the child was not cooperative and refused to wear a surgical mask. To manage this case, we equipped the aunt with an N-95 mask and face shield during transfer to the red zone, and the child was covered with an aerosol tube delivery system to minimize contamination. We also opted for an intravenous induction rather that the inhalational induction process which is commonly carried out in our centre for paediatrics. A light sedative was given in the admission room to avoid a crying episode and for when the child was taken out of the aerosol tube and placed onto the operating table. We also opted for rapid sequence induction, video laryngoscopy, and use of the lowest possible flow of inhalation as a standard procedure with COVID-19, as many guidelines stated [11–13]. During anaesthesia, ventilator settings, saturation, and end tidal CO_2_ were unremarkable. Postoperatively, we gave the child an extra opioid bolus to avoid buckling or coughing and to avoid further aerosol formation.

Interestingly, we found our patient's Ct interval decreasing after general anaesthesia. Some studies used the cycle threshold (Ct) value as a semiquantitative measure of the SARS-CoV-2 viral load, which was the highest around symptom onset, steadily decreasing during the first 10 days after illness onset and then plateauing. In the first week after symptom onset (days 2 to 7), the geometric mean (GM) of Ct was 28.18 (95% confidence interval (CI): 27.76–28.61). In the second week (days 8 to 14), the Ct was 30.65 (95% CI: 29.82–31.52; *p* < 0.001 compared with week 1), and after 14 days, the GM Ct was 31.60 (95% CI: 31.60–34.49; *p* = 0.01 compared with week 1). There was no significant difference in Ct values between days 8 and 14 and after 14 days (*p* = 0.49) [14]. This probability made us presume that general anaesthesia lowers immunity and then introduces the virus from its nasopharyngeal pocket to the lungs via endotracheal intubation which may possibly be linked to an increase in the viral load of the patient. The correlation between general anaesthesia, the immune reaction to COVID-19, and mortality would need further investigation in the paediatric population, although similar studies on adult patients have seen an increase in morbidity and mortality [14, 15].

The child had a longer hospital stay due to her COVID-19 status compared to a similar non-COVID-19 case where the patient was discharged the next day, and she may potentially infect her aunt who had to stay to care for her in isolation. After the procedure, the entire surgical team continued to provide medical services while having to follow hospital policy and to report any symptoms. They also had to have twice daily temperature checks using an online monitoring platform. At the end of 14 days of observation by the hospital infection prevention team, all the team members were cleared from observation with none having been infected.

## 4. Conclusions

Safe paediatric anaesthesia, especially in high-risk aerosol-generating procedures of paediatric patients, in the COVID-19 era requires careful consideration and preparation of both the patient and the healthcare providers. Multidisciplinary team work with an emphasis on a systematic and planned approach is required to improve efficiency.

## Figures and Tables

**Table 1:** Patient real-time polymerase chain reaction results for SARS-CoV-2.

Date		RT-PCR result	Details (kit: MbioCoV)
31/10	Preop	Positive	(Ct) helicase ORF1ab 33.74 gen RDRP 36.98
2/11	Postop day 2	Positive	(Ct) helicase ORF1ab 26.06 gen RDRP 27.24
7/11	Postop day 7	Positive	(Ct) helicase ORF1ab 21.7 gen RDRP 23.71
14/11	Postop day 14	Positive	(Ct) helicase ORF1ab 28.1 gen RDRP 30.28
18/11	Postop day 18	Positive	(Ct) helicase ORF1ab 34.58 gen RDRP 38.25
23/11	Postop day 23	Negative	—
24/11	Postop day 24	Negative	—
